# circ_0082375 promotes the progression of glioma by regulating Wnt7B

**DOI:** 10.1515/tnsci-2020-0181

**Published:** 2021-11-19

**Authors:** Xianbing Meng, Hailong Tian, Wenqiang Guo, Zhigang Wang

**Affiliations:** Department of Neurosurgery, Qilu Hospital of Shandong University, No. 758, Hefei Road, Shibei District, Qingdao City, Shandong Province, 266035, China; Department of Neurosurgery, The Second Affiliated Hospital of Shandong First Medical University, Tai’an, 271000, China

**Keywords:** glioma, circ_0082375, miR-485-5p, Wnt7B

## Abstract

Circular RNAs contribute to the progression of glioma. However, the biological role and underlying mechanism of circ_0082375 in glioma remain unclear. Quantitative real-time PCR and Western blot assay were used to evaluate the expression levels of circ_0082375, microRNA-485-5p, and Wnt family member 7B (Wnt7B). The overall survival of glioma patients was estimated by the Kaplan–Meier curve. Cell proliferation, apoptosis, invasion, and migration were detected by cell counting kit-8, 5-ethynyl-2 -deoxyuridine (EdU) staining, flow cytometry, and transwell assays, respectively. Glucose level and lactate production were determined using glucose and lactate assay kits. *In vitro* angiogenesis assay was used to evaluate the angiogenesis of glioma cells. The interaction between microRNA (miR)-485-5p and circ_0082375 or Wnt family member 7B (Wnt7B) was verified by dual-luciferase reporter and RNA immunoprecipitation assays. A xenograft model was used to verify the function of circ_0082375 *in vivo*. circ_0082375 was upregulated in glioma tissues, and it was closely related to the prognosis of glioma patients. circ_0082375 knockdown suppressed cell proliferation, migration, invasion, angiogenesis, glycolysis, and epithelial-mesenchymal transition (EMT), and promoted cell apoptosis in glioma cells. irc_0082375 was a sponge of miR-485-5p, which directly targeted Wnt7B. Knockdown of circ_0082375 inhibited the malignancy, angiogenesis, and glycolysis of glioma cells *in vitro* by sponging miR-485-5p. Besides, circ_0082375 knockdown hampered the growth of glioma growth by regulating the miR-485-5p/Wnt7B axis *in vivo.* Altogether, circ_0082375 regulated miR-485-5p/Wnt7B axis to promote the malignancy, angiogenesis, and glycolysis of glioma cells, thereby contributing to the progression of glioma.

## 1 Introduction

Glioma is a common primary intracranial tumor with strong invasiveness [1]. It was reported that the morbidity of glioma has risen from 5.9 per 100,000 people in 1973 to 6.61 per 100,000 people in 2016 [2]. Although significant progress has been made in surgical and chemotherapy techniques, glioma remains one of most frequent causes of cancer deaths globally [3]. Thus, seeking a novel biomarker for the treatment of glioma patients is urgently required.

Circular RNAs (circRNAs), which are novel RNA molecules formed by covalently closed loops, have received a lot of attention [4]. Interestingly, circRNAs partake in the regulation of multiple pathological processes in glioma, containing EMT, proliferation, apoptosis, migration, invasion, angiogenesis, and glycolysis [5,6]. For instance, circRNA pleiotrophin facilitates glioma cell proliferation and stemness by interacting with miR-145-5p and miR-330-5p [7]. Furthermore, circPITX1 reduces radiation sensitivity of glioma cells by elevating glycolysis through regulating the miR-329-3p/NIMA2 axis [8]. hsa_circ_0082375 (chr7: 129950626–129964020), derived from the carboxypeptidaseA4 (*CPA4*) gene, has been uncovered to be upregulated in glioma tissues of 73 patients [9], whereas the underlying mechanism of hsa_circ_0082375 in glioma has not been clearly explained yet.

MiRNAs are partly responsible for the progression and tumorigenesis of glioma cells [10]. Several miRNAs, such as miR-504 [11], miR-150 [12], and miR-1265 [13], have been proved to be unconventionally expressed in glioma and are implicated in glioma progression by affecting cell growth, EMT, and glycolysis. MiR-485-5p has lower levels in a variety of cancers [14], and it plays an antitumor role in oral squamous cell carcinoma [15] and esophageal cancer [16]. This study validates the underlying mechanism of miR-485-5p in glioma cells.

WNT signaling is one of the most vital pathways for tumor development [17]. Among these genes, myeloid Wnt7B exerts a promoting effect on cell invasion, angiogenesis, and lung metastasis in breast cancer [18]. Importantly, Wnt7B regulates cell proliferation and invasion in glioma [19]. Nevertheless, the effects of Wnt7B on glioma cell malignancy, angiogenesis, and glycolysis need to be further studied.

Here, we aimed to investigate the function of circ_0082375 in glioma. Besides, the impact of the circ_0082375/miR-485-5p/Wnt7B axis on glioma progression was enquired, providing an effective molecular target for glioma therapy.

## 2 Materials and methods

### 2.1 Tissue specimens and cell

Tumor specimens were obtained from 48 glioma patients who underwent surgery at Qilu Hospital of Shandong University. Normal brain tissues were acquired from 48 patients without glioma who experienced partial brain resection. None of the participants received treatment before surgery.

Human glioma cell lines U-251 (CL-0237, Procell, Wuhan, China) and LN-229 (CRL-2611, American Tissue Culture Collection, Manassas, VA, USA). All cells were cultivated in Dulbecco’s modified Eagle’s medium (Gibco, Carlsbad, CA, USA) with 10% fetal bovine serum (FBS, Gibco). The incubator for cell growth was filled with 5% CO_2_ and kept at 37°C.


**Informed consent:** Informed consent has been obtained from all individuals included in this study.
**Ethical approval:** The research related to human use has been complied with all the relevant national regulations, institutional policies and in accordance the tenets of the Helsinki Declaration and has been approved by the Ethics Committee of Qilu Hospital of Shandong University.

### 2.2 Transfection

Full-length sequences of circ_0082375 and Wnt7B (NM_058238) were inserted into the empty pCD5-ciR and pcDNA vectors (GenePharma, Shanghai, China) to produce circ_0082375 and Wnt7B overexpressed plasmids, respectively. Small interfering RNAs against circ_0082375 (si-circ_0082375#1, si-circ_0082375#2, and si-circ_0082375#3), miR-485-5p mimics (miR-485-5p) and inhibitor (anti-miR-485-5p), and short-hairpin RNA plasmid against circ_0082375 (sh-circ_0082375) and their matched controls (si-NC, NC, anti-NC, and sh-NC) were synthesized by RiboBio (Guangzhou, China). All sequences were exhibited in Table 1. Lipofectamine^®^ 3000 (Invitrogen, Carlsbad, CA, USA) was used for the execution of transfection when cell density reached 60–70%.

**Table 1:** Oligonucleotide sequences used in this study

Oligonucleotide name	5'–3'
si-circ_0082375#1	TCCCTTTCTGAAACTCACATT
si-circ_0082375#2	CTTTCTGAAACTCACATTGAA
si-circ_0082375#3	GGCTCCCTTTCTGAAACTCAC
si-NC	GAGTCTCGTTGCGTTGTAATGATCA
miR-485-5p	AGAGGCUGGCCGUGAUGAAUUC
NC	UCACAACCUCCUAGAAAGAGUAGA
anti-miR-485-5p	GAATTCATCACGGCCAGCCTCT
anti-NC	CAGUACUUUUGUGUAGUACAA

### 2.3 RNA isolation and RNase R treatment

Total RNA was extracted from glioma tissues and cells using TRIzol reagent (Invitrogen). Cytoplasmic and nuclear RNA purification kit (Amyjet Scientific, Wuhan, China) was used to isolate RNA from the cytoplasmic and nuclear RNA fraction following the manufacturer’s instructions.

For RNase R digestion, 3 μg RNA was incubated with or without RNase R (3 U/µg, Epicentre, Illumina, Inc.) at 37°C for 30 min, followed by quantitative real-time PCR (RT-qPCR) analysis, as described previously [20].

### 2.4 RT-qPCR

The cDNA was extracted by using PrimeScript^TM^ RT reagent kit (TaKaRa, Wuhan, China) or miScript II RT Kit (TaKaRa). qPCR was performed using a SYBR premix Ex TaqII kit (TaKaRa) on 7500 Real-Time PCR System (Applied Biosystems, Foster City, CA, USA). The primers used for RT-qPCR were shown in Table 2. GAPDH and U6 were used as the internal controls.

**Table 2:** Primer sequences for RT-qPCR

Gene name	Forward primer (5'–3')	Reverse primer (5'–3')
circ_0082375	ACAGCATCTGGTGTGTGCTT	TTTCACCTCCACTTCCGAAT
CPA4	TGGATGTCCTGGTCCCATCT	GGGAATGGTAAGCCCCGTAG
18S rRNA	GTGGTGTTGAGGAAAGCAGACA	TGATCACACGTTCCACCTCATC
miR-485-5p	CGAGAGGCTGGCCGTGAT	GTCGTATCCAGTGCAGGGTCCGAGGTATTCGCACTGGATACGACGAATTC (RT)
U6	CGCTTCGGCAGCACATATAC	TTCACGAATTTGCGTGTCATC
Wnt7B	AGAAGACCGTCTTCGGGCAAGA	AGTTGCTCAGGTTCCCTTGGCT
GAPDH	GAGAGAAACCCGGGAGGCTA	GCGCCCAATACGACCAAATC

### 2.5 Cell counting kit-8 (CCK-8) assay

The glioma cells were added into 96-well plates. At 24, 48, and 72 h posttransfection, CCK-8 kit (Solarbio, Beijing, China; 10 μL/well) was incubated with the cells at 37°C for 2 h. The absorbance was calculated at 450 nm by Biotek-Epoch2 (Beijing, China).

### 2.6 EdU staining assay

EdU staining was conducted using a Cell-Light EdU Apollo643 *In Vitro* Kit (Riobio, Guangzhou, China). Briefly, transfected cells were plated for 24 h in a 96-well plate. Then cells were incubated with 50 μM EdU solution for 2 h. After stained with a nuclear dye Hoechst 33342 for 30 min, cell proliferation was estimated using a fluorescence microscope (Thermo Fisher Scientific, Rockville, MD, USA).

### 2.7 Flow cytometry assay

The transfected U-251 and LN-229 cells were plated in a six-well plate for 48 h. Then cells were incubated with Annexin V fluorescein isothiocynate and propidium iodide for 20 min based on the manufacturer’s instructions. Cell apoptosis was analyzed on a BD-FACS Canto II (BD, San Jose, CA, USA).

### 2.8 Transwell assay

Transwell assay was utilized to check cell migration and invasion. The only difference was that the chamber was precoated with Matrigel (Corning Incorporated, Corning, New York, USA) when the transwell invasion assay was performed. The transfected U-251 and LN-229 cells were digested, and 200 μL of cell suspension was inoculated into the upper chamber supplemented with serum-free medium. The lower chamber was added with 600 μL of culture medium with 10% FBS. About 36 h later, the cells were stained with crystal violet (Sigma-Aldrich, St. Louis, MO, USA) for 20 min. The results were photographed using a microscope and counted using Image-Pro Plus v6.0 software (Media Cybernetics, Inc., Bethesda, MD, USA).

### 2.9 *In vitro* angiogenesis assay

An *in vitro* angiogenesis assay kit (Invitrogen, Carlsbad, CA, USA; Chuan Qiu Biotechnology, Shanghai, China) was used for *in vitro* angiogenesis assay. Briefly, ECMatrix™ was placed into each well of a 96-well plate. Human umbilical vein endothelial cells (HUVEC) were precultured in a serum-free medium for 12 h. Then HUVEC cells and U-251 or LN-229 cells were transferred onto the surface of the polymerized ECMatrix™ and allowed to coculture for 16 h. Then tube formation was evaluated under a microscope and analyzed using Image-Pro Plus v6.0 software(Media Cybernetics).

### 2.10 Glucose level and lactate production detection

U-251 and LN-229 cells were transfected and further incubated for 48 h. The supernatant of the cell medium was then collected, and the levels of glucose and lactate were estimated by a glucose and lactate assay kit (Sigma-Aldrich). Lactate production was calculated by subtracting the concentration of lactate in the primordial medium from the concentration of lactate in the collected supernatant.

### 2.11 Western blot assay

The proteins of glioma cells and tissues were extracted using radio-immunoprecipitation assay (Sigma-Aldrich) and denatured at 100°C before they were separated using sodium dodecyl sulphate-polyacrylamide gel electrophoresis. After transferring to the polyvinylidene fluoride (Sigma-Aldrich) membranes and immersing in 5% skimmed milk, the membranes were conjugated with primary antibodies against Wnt7B (ab94915, 1:1,000, Abcam, Cambridge, MA, USA), Total-caspase 3 (ab32351, 1:5,000), cleaved-caspase 3 (ab32042, 1:5,000), N-cadherin (ab76057, 1:1,500), E-cadherin (ab1416, 1:100), fibronectin (FN, ab45688, 1:1,000), and snail (ab180714, 1:1,000), proliferating cell nuclear antigen (PCNA) (ab18197, 1:2,000), or GAPDH (ab8245, 1:2,000) at 4°C overnight. Following, the membranes were mixed with corresponding secondary antibodies (ab6721, 1:20,000; ab6789, 1:15,000) for 1 h. Finally, an enhanced chemiluminescence Western blotting system (Bio-Rad, Hercules, CA, USA) was used to detect these membranes.

### 2.12 RNA immunoprecipitation (RIP) assay

RIP assay was performed by using a Magna RIP™ RNA-binding protein kit (Millipore, Billerica, MA, USA). U-251 and LN-229 cells were lysed, and the collected supernatants were incubated with magnetic beads precoated with Ago2 antibody or IgG antibody overnight at 4°C. Then RNAs were purified and extracted by TRIzol (Invitrogen), followed by the estimation of the abundance of circ_0082375 or Wnt7B and miR-485-5p using RT-qPCR analysis.

### 2.13 Dual-luciferase reporter assay

The wild-type (wt) and mutant type (mut) sequences of circ_0082375 and Wnt7B 3′-untranslated region (3′-UTR) with miR-485-5p-binding sites were inserted into the pmirGLO vector (Promega, Madison, WI, USA), named as circ_0082375-wt, Wnt7B-wt, circ_0082375-mut, or Wnt7B-mut. The constructed luciferase plasmids were cotransfected with control plasmid and miR-485-5p or NC into U-251 and LN-229 cells before the cell density reached 60–70%. About 48 h later, luciferase activity was estimated by a dual-luciferase reporter gene assay kit (Promega).

### 2.14 Xenograft model

The animal experiments were executed with the approval of the Animal Research Committee of Qilu Hospital of Shandong University. U-251 cells with stably expressing sh-circ_0082375 or sh-NC were injected into 6-week-old male nude mice (*n* = 5 per group), which were purchased from Beijing Vital River Laboratory Animal Technology Co., Ltd. (Beijing, China). Tumor volume was measured once a week. Five weeks after injection, all mice were euthanized using pentobarbital sodium, and tumor tissues were gathered and weighed. In addition, tumor tissues were stored in liquid nitrogen for further examination.

### 2.15 Statistical analysis

GraphPad Prism V6.0 (GraphPad Software, Inc, San Diego, CA, USA) was used to perform the statistical analysis. Data were represented as the mean ± standard deviation (SD). For each expression analysis, three biological replicates were performed. All results were inspected using Student’s *t*-test (two groups) or one-way ANOVA (multiple groups). *P* < 0.05 was regarded as statistically significant.

## 3 Results

### 3.1 circ_0082375 is elevated in glioma tissues and cells

**Figure 1:**
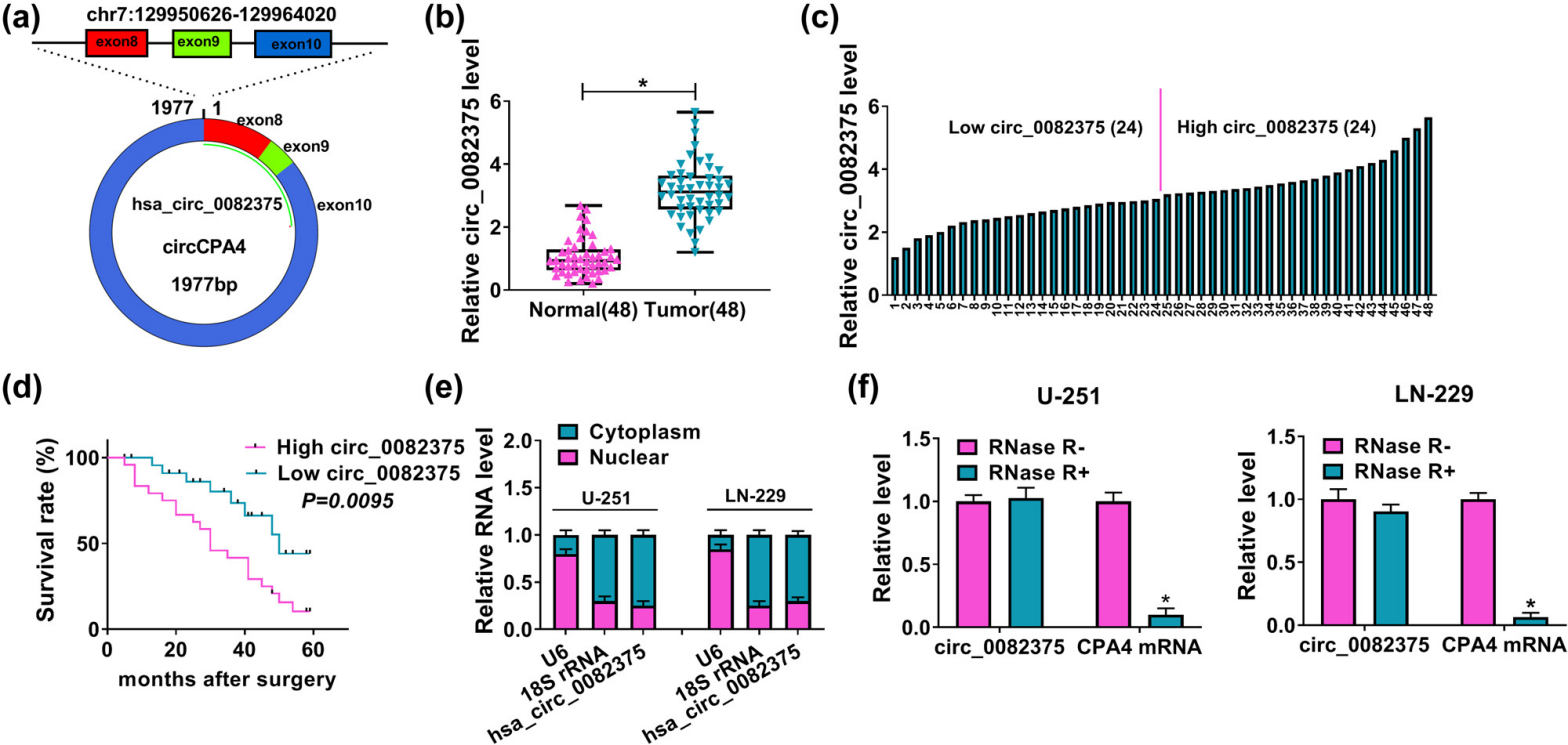
circ_0082375 is highly expressed in glioma tissues and cells. (a) Schematic representing the formation of circ_0082375. (b) circ_0082375 levels were measured by RT-qPCR in glioma tissues. *n* = 48 per group. (c) The 48 cases of glioma patients were divided into low circ_0082375 group (*n* = 24) and high circ_0082375 group (*n* = 24) based on the median value of circ_0082375 expression. (d) The overall survival rate of glioma patients with high (*n* = 24) or low (*n* = 24) circ_0082375 expression was evaluated using Kaplan–Meier survival curve analysis. (e) Nuclear-cytoplasmic separation experiment was performed to evaluate the cellular localization of circ_0082375 in glioma cells (U-251 and LN-229 cells). U6 was used as a positive control for nucleus, whereas 18 S rRNA was used as a positive control for cytoplasm. *n* = 3 for each group. (f) Relative levels of circ_0082375 and linear CPA4 in glioma cells with or without RNase R digestion were analyzed by RT-qPCR. *n* = 3 for each group. All data were presented as the mean ± SD from three independent experiments. Statistical significance was determined using Student’s *t* test. *P < 0.05.

hsa_circ_0082375 (chr7: 129950626-129964020), termed circCPA4, is derived from circularization of exon8, exon9, and exon10 of the *CPA4* gene (Figure 1a). Figure 1b displayed that circ_0082375 was aberrantly elevated in glioma tissues (*n* = 48) in contrast to 48 cases of normal tissues. Then the 48 cases of glioma patients were divided into high circ_0082375 group (*n* = 24) and low circ_0082375 group (*n* = 24) based on the median value of circ_0082375 expression (Figure 1c). And Kaplan–Meier curves uncovered that patients with high circ_0082375 expression group had worse overall survival (*P* = 0.0095) than those glioma patients with low circ_0082375 expression (Figure 1d). Besides, circ_0082375 was preferentially distributed in in the cytoplasm of glioma cells (Figure 1e), indicating that circ_0082375 might act as a miRNA sponge in the cytoplasm. Furthermore, RNase R treatment followed by RT-qPCR assay indicated that circ_0082375, but not its linear transcript CPA4, was resistant to RNase R digestion (Figure 1f). The above results uncovered that circ_0082375 was inextricably linked to the prognosis of glioma patients.

### 3.2 circ_0082375 knockdown induces apoptosis and impedes proliferation, migration, invasion, angiogenesis, glycolysis, and EMT in glioma cells

**Figure 2:**
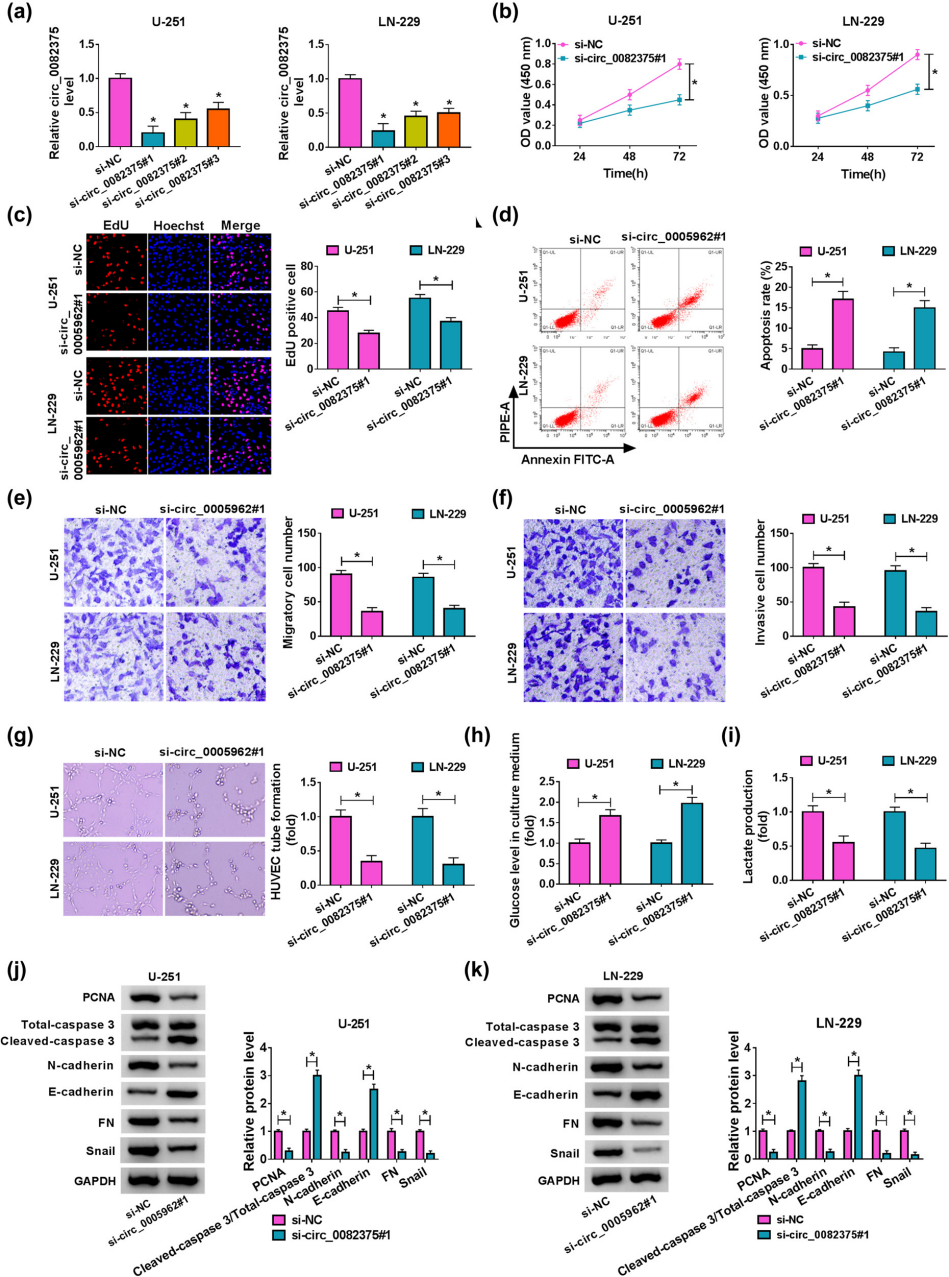
circ_0082375 knockdown induces cell apoptosis and inhibits cell proliferation, migration, invasion, angiogenesis, glycolysis, and EMT in glioma cells. (a and b) circ_0082375 expression in glioma cells transfected with three specific siRNA against circ_0082375 (circ_0082375#1, circ_0082375#2, and circ_0082375#3) was determined by RT-qPCR. (b and c) CCK-8 and EdU analysis of cell proliferation in glioma cells transfected with si-circ_0082375#1 or si-NC. (d) The apoptosis of glioma cells with si-circ_0082375#1 or si-NC transfection was analyzed by flow cytometry assay. (e and f) Cell migration and invasion of glioma cells with si-circ_0082375#1 or si-NC transfection were evaluated by transwell assay. (g) Matrigel-based tube formation assay was performed to assess the *in vitro* angiogenesis ability of glioma cells with si-circ_0082375#1 or si-NC transfection. (h and i) Glucose level and lactate production in glioma cells with si-circ_0082375#1 or si-NC transfection were determined. (j and k) The protein levels of PCNA, cleaved-caspase 3, total-caspase 3, N-cadherin, E-cadherin, FN, and snail in glioma cells transfected with si-circ_0082375#1 or si-NC were determined by Western blot. All data were presented as the mean ± SD from three independent experiments. Statistical significance was determined using Student’s *t* test. *P < 0.05.

For investigation of circ_0082375 function in glioma, siRNA against circ_0082375 were transfected into U-251 and LN-229 cells. As displayed in Figure 2a, circ_0082375 was downregulated in glioma cells after si-circ_0082375#1, si-circ_0082375#2, or si-circ_0082375#3 transfection. Si-circ_0082375#1 was used for further investigation due to its high knockdown efficiency. Inhibition of circ_0082375 hampered the proliferation of U-251 and LN-229 cells, as the OD value (Figure 2b) and EdU-positive cells (red; Figure 2c) are decreased in cells with circ_0082375 knockdown, in contrast with the si-NC group. Flow cytometry assay uncovered that knockdown of circ_0082375 elevated the apoptosis rate of glioma cells (Figure 2d). Besides, cell migration and invasion were also curbed in cells with circ_0082375 knockdown (Figure 2e and f). Furthermore, circ_0082375 knockdown decreased the angiogenesis of HUVEC cells cocultured with glioma cells (Figure 2g). In addition, the elevated levels of glucose in the culture medium (Figure 2h) and the decreased levels of lactate production (Figure 2i) in cells with circ_0082375 knockdown, suggesting that the glycolysis of glioma cells was inhibited by circ_0082375 knockdown. Furthermore, the protein levels of proliferative marker PCNA, apoptosis marker cleaved-caspase 3, and EMT markers containing E-cadherin, N-cadherin, FN, and snail were detected. As shown in Figure 2j and k, PCNA protein levels were elevated while the ratio of cleaved-caspase 3/total-caspase 3 was elevated in glioma cells with circ_0082375 knockdown. Besides, circ_0082375 knockdown inhibited EMT phenotypes in glioma cells, with the upregulation of E-cadherin and the downregulation of N-cadherin, FN, and snail (Figure 2j and k). These results uncovered that circ_0082375 promotes glioma cell progression by regulating proliferation, apoptosis, migration, invasion, angiogenesis, glycolysis, and EMT.

### 3.3 circ_0082375 functions as a molecule sponge for miR-485-5p

**Figure 3:**
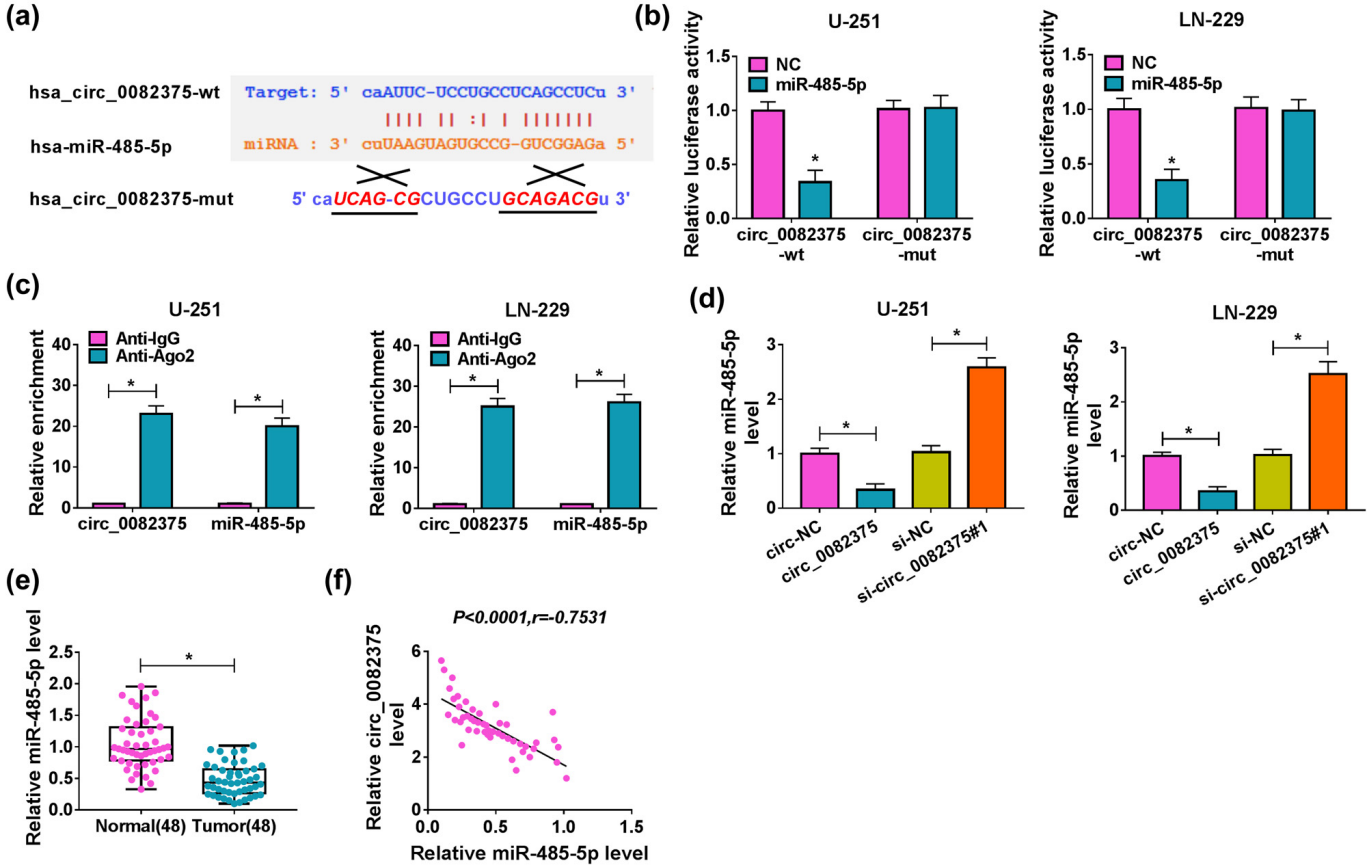
circ_0082375 acts as an endogenous sponge for miR-485-5p. (a) The binding sites between circ_0082375 and miR-485-5p were predicted by StarbaseV3.0. (b) Relative luciferase activity of glioma cells cotransfected with recombinant luciferase reporter plasmids (circ_0082375-wt and circ_0082375-mut) and miR-NC or miR-485-5p. (c) Ago2 RIP assay was performed to verify the correlation between circ_0082375 and miR-485-5p. (d) The expression of miR-485-5p in glioma cells transfected with circ-NC, circ_0082375, si-NC, or si-circ_0082375#1 was detected by RT-qPCR. (e) The expression of miR-485-5p in glioma tissues (*n* = 48) and normal tissues (*n* = 48) was detected by RT-qPCR. (f) Pearson correlation coefficient was used to evaluate the correlation between the expression levels of circ_0082375 and miR-485-5p in glioma tissues (*n* = 48). All data were presented as the mean ± SD from three independent experiments. Statistical significance was determined using Student’s *t* test. *P < 0.05.

CircRNAs are reported to perform their biological functions by serving as competing endogenous RNAs or endogenous sponge for miRNAs [21]. Bioinformatics software Starbase v2.0 (http://starbase.sysu.edu.cn/) database showed that miR-485-5p might interact with circ_0082375 (Figure 3a). For the sake of confirmation, we constructed the wild-type (circ_0082375-wt) and mutant-type (circ_0082375-mut) reporter plasmids of circ_0082375. Figure 3b disclosed that transfection of miR-485-5p mimic obviously decreased the luciferase activity of the circ_0082375-wt group rather than the circ_0082375-mut group, compared with the cells with NC transfection. Besides, circ_0082375 and miR-485-5p were significantly gathered in cells incubated with anti-Ago2 antibody in contrast with cells incubated with anti-IgG antibody (Figure 3c). Thus, we confirmed that circ_0082375 served as a miR-485-5p sponge. Furthermore, the levels of miR-485-5p in U-251 and LN-229 cells were downregulated by circ_0082375 overexpression and upregulated by circ_0082375 silencing (Figure 3d). In addition, miR-485-5p expression in 48 cases of glioma tissues was noticeably decreased compared to normal tissues (*n* = 48; Figure 3e), and a negative correlation (*r* = −0.7531, *P* < 0.0001) was found between circ_0082375 and miR-485-5p expression in 48 cases of glioma tissues (Figure 3f). Altogether, circ_0082375 acted as a molecule sponge for miR-485-5p.

### 3.4 Knockdown of miR-485-5p rescues circ_0082375 silencing-mediated suppression effects on the progression of glioma cells

**Figure 4:**
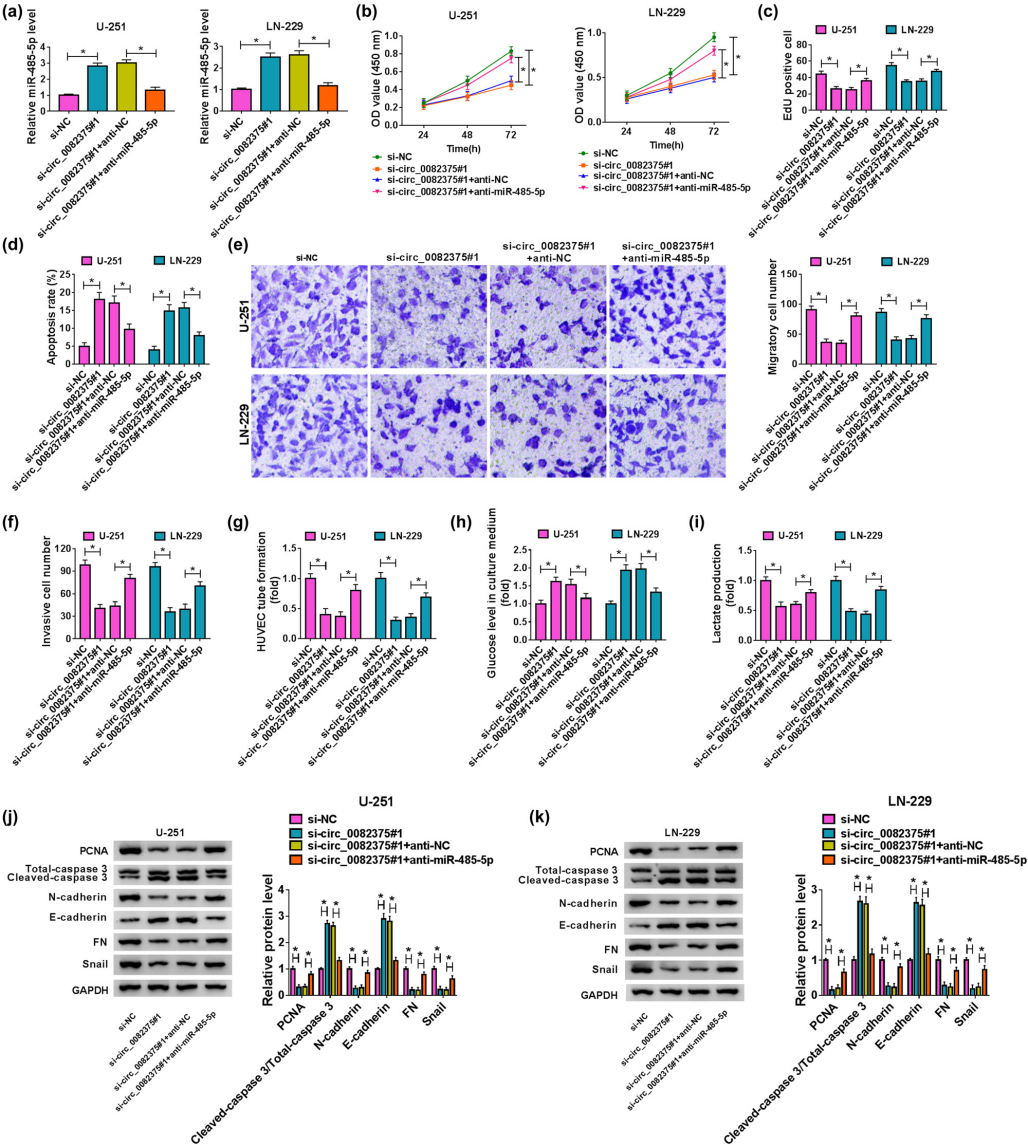
Knockdown of miR-485-5p partly reverses the effect of circ_0082375 inhibition on the progression of glioma. U-251 and LN-229 cells were transfected with si-NC, si-circ_0082375#1, si-circ_0082375#1 + anti-NC, or si-circ_0082375#1 + anti-miR-485-5p, respectively. (a) The expression of miR-485-5p in transfected glioma cells was examined by RT-qPCR. (b and c) The proliferation of transfected cells was analyzed by CCK-8 and EdU assay. (d) Flow cytometry was used to analyze the apoptosis of transfected cells. (e and f) Transwell assays were used to detect cell migration and invasion. (g) *In vitro* angiogenesis ability of glioma cells was detected by Matrigel-based tube formation assay. (h and i) Glucose level and lactate production in U-251 and LN-229 cell culture medium were assessed. (j and k) Western blot assay was used to measure the protein levels of PCNA, cleaved-caspase 3, total-caspase 3, N-cadherin, E-cadherin, FN, and snail. All data were presented as the mean ± SD from three independent experiments. Statistical significance was determined using the one-way ANOVA method. *P < 0.05.

Rescue experiments were performed, and the results exhibited that miR-485-5p levels were upregulated in cells with si-circ_0082375#1 transfection, whereas this elevation was restored by cotransfection of anti-miR-485-5p (Figure 4a). Besides, miR-485-5p knockdown partly reversed the suppressive effect of si-circ_0082375#1 transfection on cell proliferation (Figure 4b and c). The promoting effect of si-circ_0082375#1 transfection on cell apoptosis (Figure 4d) and the inhibiting effects of circ_0082375 silencing on cell migration (Figure 4e), invasion (Figure 4f), angiogenesis (Figure 4g), as well as glycolysis (Figure 4h and i), were partly reversed by downregulation of miR-485-5p. And it was further confirmed that the inhibitory effects of circ_0082375 knockdown on PCNA, N-cadherin, FN, and snail protein levels, as well as the promoting effects on E-cadherin protein levels and the ratio of cleaved-caspase 3/total-caspase 3 were partly rescued by miR-485-5p inhibitor (Figure 4j and k). Thus, these findings show that circ_0082375 promotes glioma progression by negatively regulating miR-485-5p expression.

### 3.5 circ_0082375 promotes Wnt7B expression by interacting with miR-485-5p

**Figure 5:**
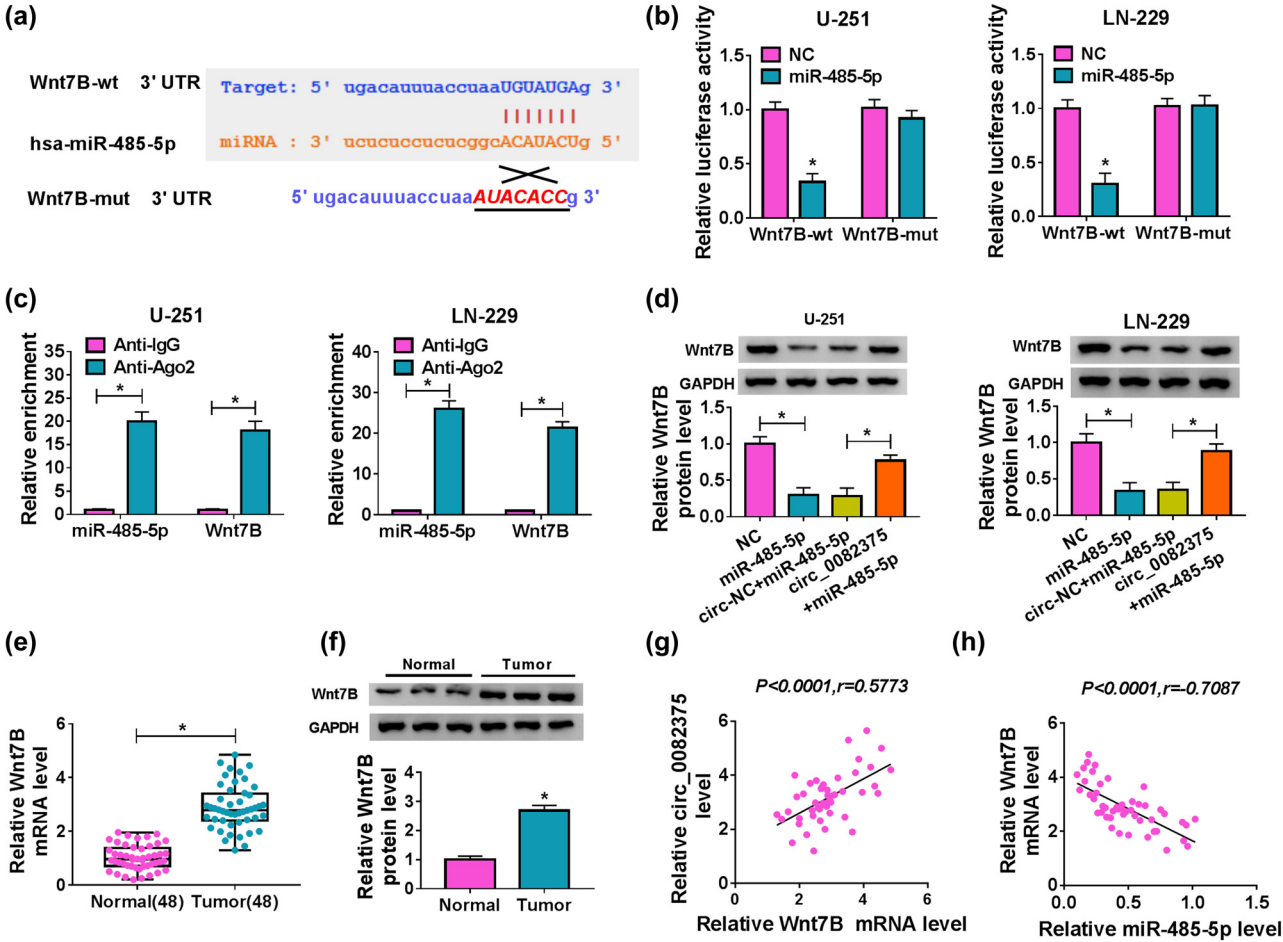
Wnt7B is negatively regulated by miR-485-5p and negatively regulated by circ_0082375. (a) The complementary binding sites between miR-485-5p and Wnt7B were predicted by StarbaseV3.0. (b and c) Dual luciferase reporter assay and RIP assay were conducted to detect the regulation between miR-485-5p and Wnt7B. (d) Wnt7B protein level in glioma cells transfected with NC, miR-485-5p, circ-NC + miR-485-5p, or circ_0082375 + miR-485-5p. (e and f) The mRNA and protein levels of Wnt7B in glioma tissues (*n* = 48) and normal tissues (*n* = 48) were detected using RT-qPCR and Western blot. (g and h) The correlation between Wnt7B and circ_0082375 or miR-485-5p in glioma tissues was evaluated by the Pearson correlation coefficient. All data were presented as the mean ± SD from three independent experiments. Statistical significance was determined using the one-way ANOVA method. *P < 0.05.

Subsequently, the downstream targets of miR-485-5p were predicted by the Starbasev2.0 database and found that Wnt7B 3'-UTR sequence harbored complementary binding sites to miR-485-5p (Figure 5a). Besides, cotransfection of miR-485-5p mimic and Wnt7B-wt restrained the luciferase activity more than half in contrast with the NC group. However, miR-485-5p mimic did not significantly affect the luciferase activity of the Wnt7B-mut group (Figure 5b). As expected, both miR-485-5p and Wnt7B were gathered by the anti-Ago2 antibody (Figure 5c). Furthermore, we observed that circ_0082375 overexpression rescued the inhibiting effect of miR-485-5p on Wnt7B protein levels in U-251 and LN-229 cells (Figure 5d). Moreover, Wnt7B mRNA and protein levels were prominently elevated in 48 cases of glioma tissues (Figure 5e and f). Furthermore, Wnt7B expression was positively correlated with circ_0082375 expression (*P* < 0.0001, *r* = 0.5773) in glioma tissues, whereas it negatively correlated with miR-485-5p expression (*P* < 0.0001, *r* = −0.7087; Figure 5g and h). Altogether, circ_0082375 regulates Wnt7B expression by sponging miR-485-5p in glioma cells.

### 3.6 Restoration of Wnt7B alleviates the effects of miR-485-5p on the progression of glioma

**Figure 6:**
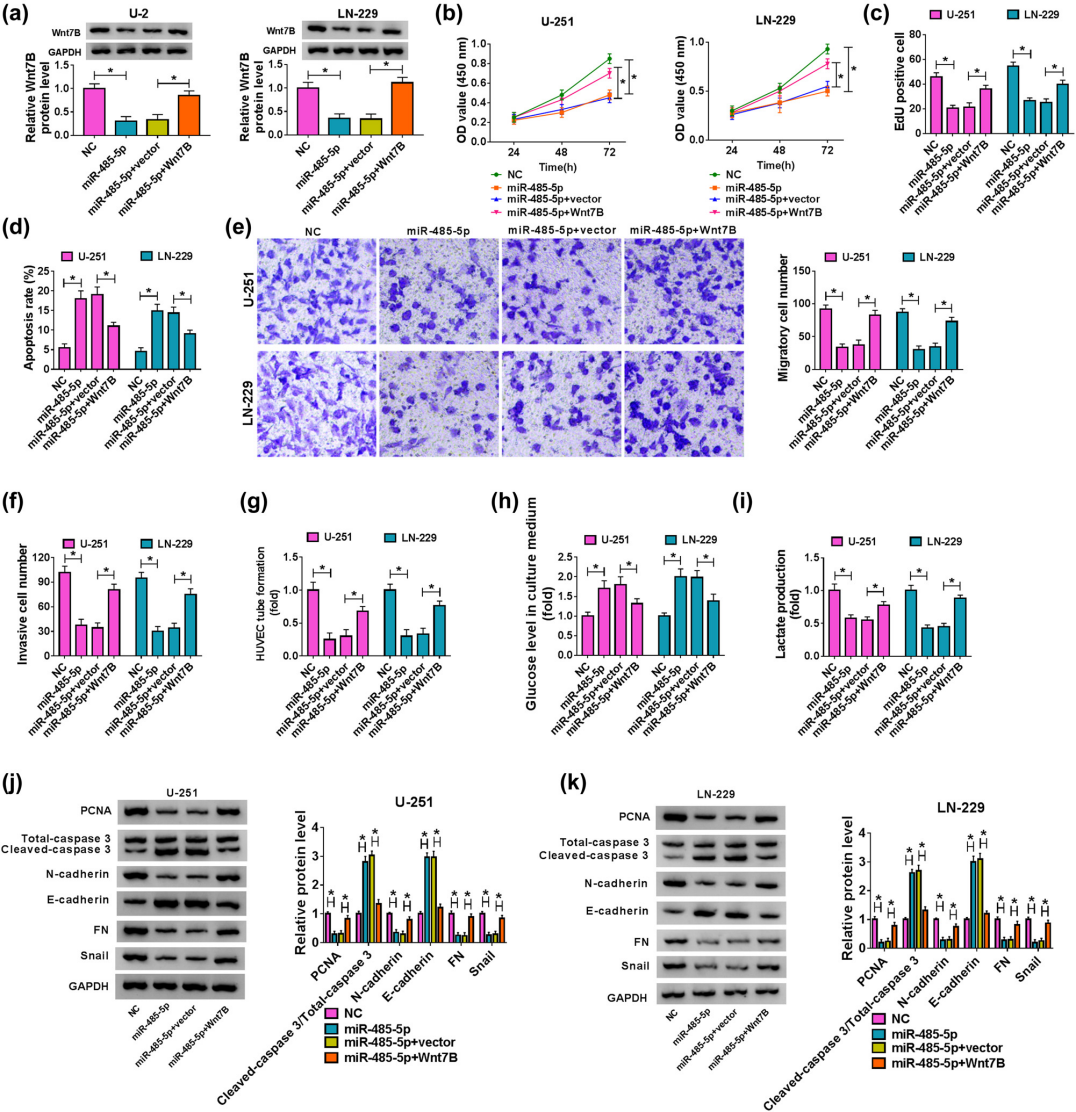
Overexpression of Wnt7B partly alleviates the effects of miR-485-5p on the progression of glioma. U-251 and LN-229 cells were transfected with NC, miR-485-5p, miR-485-5p + vector, or miR-485-5p + Wnt7B, respectively. (a) Relative protein levels of Wnt7B in transfected glioma cells were analyzed using Western blot. (b–d) CCK-8, EdU, and flow cytometry assay were conducted to analyze the proliferation and apoptosis of transfected glioma cells. (e and f) The migrated and invasive abilities of transfected glioma cells were assessed by transwell assays. (g) *In vitro* angiogenesis ability of transfected glioma cells was detected by Matrigel-based tube formation assay. (h and i) Glucose level and lactate production in the cell culture medium were detected. (j and k) The levels of PCNA, cleaved-caspase 3, total-caspase 3, N-cadherin, E-cadherin, FN, and snail protein in transfected glioma cells were determined using Western Blot. All data were presented as the mean ± SD from three independent experiments. Statistical significance was determined using the one-way ANOVA method. *P < 0.05.

U-251 and LN-229 cells were cotransfected with miR-485-5p and Wnt7B to alter Wnt7B expression and to peruse whether miR-485-5p exerts a biological role by regulating Wnt7B expression in glioma cells. Wnt7B overexpression abated the impact of miR-485-5p on Wnt7B protein levels in U-251 and LN-229 cells (Figure 6a). Moreover, miR-485-5p mimic restrained cell proliferation (Figure 6b and c), migration (Figure 6e), invasion (Figure 6f), angiogenesis (Figure 6g), and glycolysis (Figure 6h and i) but enhanced cell apoptosis (Figure 6d) in U-251 and LN-229 cells; however, these effects were partly attenuated by overexpression of Wnt7B. Furthermore, cotransfection of Wnt7B abolished the alteration in protein levels of PCNA, EMT markers, and the ratio of cleaved-caspase 3/total-caspase 3 in U-251 and LN-229 cells mediated by miR-485-5p mimic (Figure 6j and k). These findings uncovered that miR-485-5p restrains glioma development by suppressing Wnt7B expression.

### 3.7 circ_0082375 deficiency curbs the growth of glioma tumor *in vivo*

**Figure 7:**
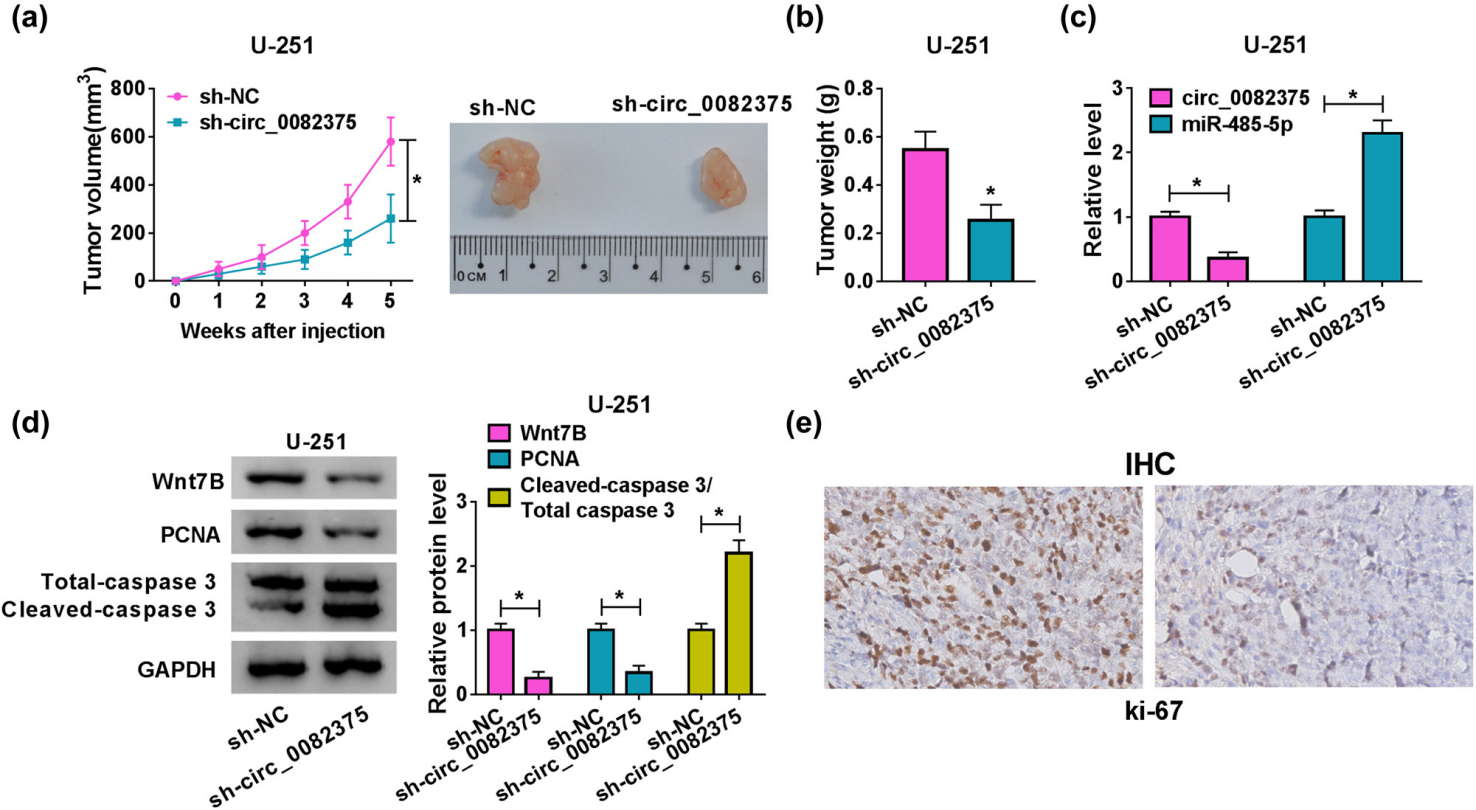
Silence of circ_0082375 suppresses the growth of glioma tumor *in vivo*. Stable U-251 cells with knockdown of circ_0082375 were subcutaneously injected into nude mice (*n* = 5 per group), and sh-NC was used as a negative control. (a) Tumor volume was measured once a week. (b) Five weeks after injection, the weight of dissected tumors was evaluated. (c) The levels of circ_0082375 and miR-485-5p in tumor tissues in sh-circ_0082375 and sh-NC groups were determined by RT-qPCR. (d) The protein levels Wnt7B, PCNA, cleaved-caspase 3, and total-caspase 3 were examined using Western Blot in tumor tissues in sh-circ_0082375 and sh-NC groups. (e) Immunohistochemistry analysis of Ki-67 in tissue sections in sh-circ_0082375 and sh-NC groups. All data were presented as the mean ± SD from three independent experiments. Statistical significance was determined using Student’s *t* test. *P < 0.05.

For investigation of the function of circ_0082375 *in vivo*, xenograft tumor models were established by injecting U-251 cells stable expressing sh-NC or sh-circ_0082375 (five mice in each group). In contrast with the sh-NC group, the tumor volume and tumor weight were declined in the sh-circ_0082375 group (Figure 7a and b). Besides, RT-qPCR assay revealed that circ_0082375 was downregulated, whereas the miR-485-5p level was upregulated (Figure 7c) in tumor tissues derived from nude mice injected with U-251 cells carrying sh-irc_0082375. Furthermore, the protein levels of Wnt7B and PCNA were declined, whereas the ratio of cleaved-caspase 3/total-caspase 3 was elevated (Figure 7d) in tumor tissues from the sh-irc_0082375 group. Furthermore, immunohistochemistry with anti-ki-67 antibody showed that circ_0082375 knockdown markedly reduced the proliferation of glioma cells (Figure 7e). These results demonstrated that interference of circ_0082375 efficiently impedes the growth of glioma *in vivo*.

## 4 Discussion

Mounting evidence suggests that circRNAs are partly responsible for the tumorigenesis and progression of glioma. circ_0082375 (hsa_circCPA4_007), derived from the *CPA4* gene, was first identified to be significantly increased in glioma by high-throughput circRNA microarray assay. And circ_0082374 (hsa_circCPA4_006) has been reported to accelerate cell proliferation and metastasis by adsorbing miRNA let-7 [9]. In our research, elevated circ_0082375 expression was found in 48 cases of glioma tissues, and the outcome of glioma patients was closely related to the expression level of circ_0082375.

Unlike normal cells that rely on mitochondrial oxidation to produce energy, tumor cells mainly generate metabolic energy by glycolysis [22]. In addition, the intermediates produced during glycolysis contribute to the growth of tumor cells [23]. Besides, angiogenesis exerts a pivotal role in glioma, as new blood vessels are indispensable for the survival and progress of glioma and can transport the necessary oxygen, growth factors, and nutrients [24]. Furthermore, research have disclosed that angiogenesis exhibits a closely intertwined with the outcome of glioma patients [25,26]. In our research, circ_0082375 knockdown inhibited cell proliferation, migration, invasion, angiogenesis, EMT, and impaired cell glycolysis and promoted cell apoptosis in glioma cells. Altogether, circ_0082375 served as a tumor promoter in glioma *in vitro*.

Emerging evidence reveals that some circRNAs located in the cytoplasm can interact with miRNAs through miRNA response elements [27]. In this study, circ_0082375 was mainly located in the cytoplasm of glioma cells, suggesting that circ_0082375 might function as a miRNA sponge. Through luciferase assay and RIP assay proved that circ_0082375 acted as a miR-485-5p sponge. A previous study revealed that MiR-485-5p curbed cell proliferation and motility in glioma [28]. In accordance with the previous research, miR-485-5p was downregulated in glioma tumors. Furthermore, repression of miR-485-5p could abolish the inhibiting impacts of circ_0082375 silencing on cell progression, migration, invasion, angiogenesis, EMT, and glycolysis, indicating the tumor-suppressive function of miR-485-5p in glioma.

Subsequently, the downstream targets of miR-485-5p were investigated, and we authenticated that Wnt7B acted as a target of miR-485-5p. As a member of the WNT signaling pathway, Wnt7B has been found to regulate the growth of mammalian cells [29]. In accordance with previous research, Wnt7B was highly expressed in glioma tissues and cells [30,31]. Our findings disclosed that the suppressive effects of miR-485-5p mimic on cell progression, migration, invasion, angiogenesis, EMT, and glycolysis could be counteracted by overexpression of Wnt7B. Besides, the authors found that circ_0082375 upregulated Wnt7B expression by sponging miR-485-5p in glioma cells. Consistent with *in vitro* results, circ_0082375 knockdown hampered tumor growth by regulating the miR-485-5p/Wnt7B axis.

The novelty of this study was the first report of the molecular mechanism of the circ_0082375/miR-485-5p/Wnt7B axis in glioma. Unfortunately, we did not explore whether circ_0082375 can be used as a diagnostic biomarker. In addition, the function of circ_0082375 overexpression in glioma in xenograft models can be explored in the future.

Together, our results demonstrated that circ_0082375 increased Wnt7B expression via sponging miR-485-5p to regulate cell proliferation, apoptosis, migration, invasion, angiogenesis, glycolysis, and EMT, thereby affecting glioma progression. These findings strengthened our comprehension on the progression of glioma and provided a novel target for treatment of the glioma.

## References

[1] Taylor LP. Diagnosis, treatment, and prognosis of glioma: five new things. Neurology. 2010;75(18 Suppl 1):S28–32. 10.1212/WNL.0b013e3181fb3661 21041768

[2] Lu J, Zhang M, Yang X, Cui T, Dai J. MicroRNA-548c-3p inhibits T98G glioma cell proliferation and migration by downregulating c-Myb. Oncol Lett. 2017;13(5):3866–72. 10.3892/ol.2017.5870 PMC5431163 28536644

[3] Stupp R, Mason WP, van den Bent MJ, Weller M, Fisher B, Taphoorn MJ, et al. Radiotherapy plus concomitant and adjuvant temozolomide for glioblastoma. N Engl J Med. 2005;352(10):987–96. 10.1056/NEJMoa043330 15758009

[4] Jeck WR, Sorrentino JA, Wang K, Slevin MK, Burd CE, Liu J, et al. Circular RNAs are abundant, conserved, and associated with ALU repeats. RNA. 2013;19(2):141–57. 10.1261/rna.035667.112 PMC3543092 23249747

[5] Sun J, Li B, Shu C, Ma Q, Wang J. Functions and clinical significance of circular RNAs in glioma. Mol Cancer. 2020;19(1):34. 10.1186/s12943-019-1121-0 PMC7023692 32061256

[6] Hao Z, Hu S, Liu Z, Song W, Zhao Y, Li M. Circular RNAs: functions and prospects in glioma. 2019;67(1):72–81. 10.1007/s12031-018-1211-2 30460608

[7] Chen J, Chen T, Zhu Y, Li Y, Zhang Y, Wang Y, et al. circPTN sponges miR-145-5p/miR-330-5p to promote proliferation and stemness in glioma. J Exp Clin Cancer Res. 2019;38(1):398. 10.1186/s13046-019-1376-8 PMC6737709 31511040

[8] Guan Y, Cao Z, Du J, Liu T, Wang T. Circular RNA circPITX1 knockdown inhibits glycolysis to enhance radiosensitivity of glioma cells by miR-329-3p/NEK2 axis. Cancer Cell Int. 2020;20(1):1–3. 10.1186/s12935-020-01169-z PMC7071619 32190004

[9] Peng H, Qin C, Zhang C, Su J, Xiao Q, Xiao Y, et al. circCPA4 acts as a prognostic factor and regulates the proliferation and metastasis of glioma. J Cell Mol Med. 2019;23(10):6658–65. 10.1111/jcmm.14541 PMC6787466 31424161

[10] Zhou Q, Liu J, Quan J, Liu W, Tan H, Li W. MicroRNAs as potential biomarkers for the diagnosis of glioma: a systematic review and meta-analysis. Cancer Sci. 2018;109(9):2651–9. 10.1111/cas.13714 PMC6125451 29949235

[11] Liu Q, Guan Y, Li Z, Wang Y, Liu Y, Cui R, et al. miR-504 suppresses mesenchymal phenotype of glioblastoma by directly targeting the FZD7-mediated Wnt-β-catenin pathway. J Exp Clin Cancer Res. 2019;38(1):358. 10.1186/s13046-019-1370-1 PMC6697940 31419987

[12] Li SJ, Liu HL, Tang SL, Li XJ, Wang XY. MicroRNA-150 regulates glycolysis by targeting von Hippel-Lindau in glioma cells. Am J Transl Res. 2017;9(3):1058–66. PMC5375998 28386333

[13] Gao F, Du Y, Zhang Y, Ren D, Xu J, Chen D. Circ-EZH2 knockdown reverses DDAH1 and CBX3-mediated cell growth and invasion in glioma through miR-1265 sponge activity. Gene. 2020;726:144196. 10.1016/j.gene.2019.144196 31669648

[14] Wang M, Cai WR, Meng R, Chi JR, Li YR, Chen AX, et al. miR-485-5p suppresses breast cancer progression and chemosensitivity by targeting survivin. Biochem Biophys Res Commun. 2018;501(1):48–54. 10.1016/j.bbrc.2018.04.129 29678577

[15] Lin XJ, He CL, Sun T, Duan XJ, Sun Y, Xiong SJ. hsa-miR-485-5p reverses epithelial to mesenchymal transition and promotes cisplatin-induced cell death by targeting PAK1 in oral tongue squamous cell carcinoma. Int J Mol Med. 2017;40(1):83–9. 10.3892/ijmm.2017.2992 PMC5466395 28535002

[16] Han DL, Wang LL, Zhang GF, Yang WF, Chai J, Lin HM, et al. MiRNA-485-5p, inhibits esophageal cancer cells proliferation and invasion by down-regulating O-linked N-acetylglucosamine transferase. Eur Rev Med Pharmacol Sci. 2019;23(7):2809–16. 10.26355/eurrev_201904_17556 31002132

[17] Zheng D, Decker KF, Zhou T, Chen J, Qi Z, Jacobs K, et al. Role of WNT7B-induced noncanonical pathway in advanced prostate cancer. Mol Cancer Res. 2013;11(5):482–93. 10.1158/1541-7786.MCR-12-0520 PMC4141540 23386686

[18] Yeo EJ, Cassetta L, Qian BZ, Lewkowich I, Li JF, Stefater(3rd) JA, et al. Myeloid WNT7b mediates the angiogenic switch and metastasis in breast cancer. Cancer Res. 2014;74(11):2962–73. 10.1158/0008-5472.CAN-13-2421 PMC4137408 24638982

[19] Lu Y, Deng X. circ_0001730 promotes proliferation and invasion via the miR-326/Wnt7B axis in glioma cells. 2019;11(11):1335–52. 10.2217/epi-2019-0121 31304776

[20] Yu J, Xu QG, Wang ZG, Yang Y, Zhang L, Ma JZ, et al. Circular RNA cSMARCA5 inhibits growth and metastasis in hepatocellular carcinoma. J Hepatol. 2018;68(6):1214–27. 10.1016/j.jhep.2018.01.012 29378234

[21] Ashwal-Fluss R, Meyer M, Pamudurti NR, Ivanov A, Bartok O, Hanan M, et al. circRNA biogenesis competes with pre-mRNA splicing. Mol Cell. 2014;56(1):55–66. 10.1016/j.molcel.2014.08.019 25242144

[22] Vander Heiden MG, Cantley LC, Thompson CB. Understanding the Warburg effect: the metabolic requirements of cell proliferation. Science. 2009;324(5930):1029–33. 10.1126/science.1160809 PMC2849637 19460998

[23] Ganapathy-Kanniappan S, Geschwind JF. Tumor glycolysis as a target for cancer therapy: progress and prospects. Mol Cancer. 2013;12:152. 10.1186/1476-4598-12-152 PMC4223729 24298908

[24] Folkman J, Cole P, Zimmerman S. Tumor behavior in isolated perfused organs: in vitro growth and metastases of biopsy material in rabbit thyroid and canine intestinal segment. Ann Surg. 1966;164(3):491–502. 10.1097/00000658-196609000-00012 PMC1477290 5951515

[25] Tan Z, Chen K, Wu W, Zhou Y, Zhu J, Wu G, et al. Overexpression of HOXC10 promotes angiogenesis in human glioma via interaction with PRMT5 and upregulation of VEGFA expression. Theranostics. 2018;8(18):5143–58. 10.7150/thno.27310 PMC6217061 30429891

[26] Tuettenberg J, Friedel C, Vajkoczy P. Angiogenesis in malignant glioma–a target for antitumor therapy? Crit Rev Oncol Hematol. 2006;59(3):181–93. 10.1016/j.critrevonc.2006.01.004 16860996

[27] Liu J, Zhao K, Huang N, Zhang N. Circular RNAs and human glioma. Cancer Biol Med. 2019;16(1):11–23. 10.20892/j.issn.2095-3941.2018.0425 PMC6528446 31119043

[28] Yu J, Wu SW, Wu WP. A tumor-suppressive microRNA, miRNA-485-5p, inhibits glioma cell proliferation and invasion by down-regulating TPD52L2. Am J Transl Res. 2017;9(7):3336–44. PMC5553883 28804551

[29] Cho C, Smallwood PM, Nathans J. Reck and Gpr124 are essential receptor cofactors for Wnt7a/Wnt7b-specific signaling in mammalian CNS angiogenesis and blood-brain barrier regulation. Neuron. 2017;95(5):1056–73.e5. 10.1016/j.neuron.2017.08.032 28858622

[30] Zhang C, Yang X, Fu C, Liu X. Combination with TMZ and miR-505 inhibits the development of glioblastoma by regulating the WNT7B/Wnt/β-catenin signaling pathway. Gene. 2018;672:172–9. 10.1016/j.gene.2018.06.030 29906532

[31] Sun WL, Kang T, Wang YY, Sun JP, Li C, Liu HJ, et al. Long noncoding RNA OIP5-AS1 targets Wnt-7b to affect glioma progression via modulation of miR-410. Biosci Rep. 2019;39(1):BSR20180395. 10.1042/BSR20180395 PMC6328889 30498093

